# Monkeypox: Is the ‘vacated niche’ being filled?

**DOI:** 10.4102/sajid.v37i1.479

**Published:** 2022-12-12

**Authors:** Jacqueline Weyer, Lucille H. Blumberg

**Affiliations:** 1 Centre for Emerging Zoonotic and Parasitic Diseases, National Institute for Communicable Diseases, Johannesburg, South Africa

Could the monkeypox or cowpox virus emerge from its natural reservoir to become a fully human-adapted pathogen, occupying the ecological niche vacated by the eradication of smallpox? We cannot know the answer, but doubt about the possibility should be tempered by the realisation that smallpox itself must once have been a zoonosis.^1^ (p. 500)

Monkeypox (MPX), a zoonotic viral infection, presents as a febrile illness with a typical vesicular-pustular rash. The disease is rarely fatal but may be temporarily debilitating, with a few experiencing severe pain and complications observed as an exception.^2^ The monkeypox virus (MPXV) is closely related to the variola virus, the causative agent of smallpox. Monkeypox was first diagnosed in humans while enhanced surveillance for smallpox was carried out during the eradication programme.^3^ Smallpox was declared eradicated on 08 May 1980 and smallpox vaccination discontinued globally. Given the serological cross-reactivity of orthopox viruses, the effect of cessation of smallpox vaccination on the possible emergence of MPX was anticipated. Arguments on the theory of ‘vacated niches’ have been ongoing since the eradication of smallpox.^4^ The theory explains how the vacated niche left by the eradicated pathogen presents a vacuum that may be filled by pathogens which have gained a competitive advantage.

In the years following the eradication of smallpox, MPX cases remained rare. From 1970 to 1979, 47 cases of MPX were reported from five countries in West and Central Africa.^5^ More than 80% occurred in children under 10 years of age who were unvaccinated for smallpox. Monkeypox hit the news in 2003, when a MPX outbreak was reported in the United States (US).^6^ This outbreak involved the importation of rodents from West Africa for the exotic pet trade. When brought under control, an increased appreciation remained for the possible risk of similar events in the future, as well as the use of MPX as a bioweapon, not only in populations with waning smallpox immunity but also in a growing population of individuals not vaccinated for smallpox. In 2004, a list of priority MPX research needs was published and included the re-establishment and strengthening of human MPX surveillance in endemic countries; studies of the characteristics of MPX and other pox-like illnesses in patients living with HIV; ecological and natural history studies; population-based studies to define the clinical, epidemiological and ecological characteristics; and improvement of laboratory diagnosis of human MPX.^7^ Regardless of the appreciation of the possible future risks associated with MPX, it remained a neglected disease in the years that followed.

A steady rise in the number of MPX cases was reported in Africa for the past two decades, notably in the Democratic Republic of Congo (DRC) and in Nigeria (since 2017, following decades of quiescence).^5^ Many argue that the orthopox-immunity gap following cessation of smallpox vaccination is a contributing factor. It should, however, be observed that little is understood about the natural ecology of MPX, and so it is not possible to determine other factors at this time that may have been driving the emergence of the zoonotic disease in endemic countries. Since the re-emergence of MPX in Nigeria, cases in travellers returning from Nigeria were reported annually from 2018 to 2021.^8^ In May 2022, the confirmation of MPX in the United Kingdom (UK) in a traveller from Nigeria was quickly evolved, and by August 2022, more than 40 000 cases of MPX was reported from nearly 100 countries.^9,10^ Men have been affected disproportionately, notably men who have sex with men, with heightened risk in individuals with multiple partners.^10^ From June 2022 to August 2022, five cases of MPX were confirmed in South Africa, all male patients, some with a history of recent travel to Europe (National Institute for Communicable Diseases). The World Health Organization declared this outbreak a public health emergency of international concern to escalate and coordinate public health responses and scale up production of, and increase access to, countermeasures. This is because of the concern that unless the epidemic can be controlled at an early stage, the disease may remain as a widespread human-to-human transmitted infectious burden. The epidemic is fraught with complexities, including stigma associated with the unfortunate naming of the disease and the fact that currently, it is primarily a vulnerable community, subjected to many years of social injustice and discrimination, that is being affected. Given the often mild presentation of the disease, those affected may not seek medical attention, evading surveillance and the health responses intended to break chains of transmission. A scramble for access to vaccines has also ensued, and questions of equity in vaccine access are raised once again.

In retrospect, the smouldering MPX risk has taken flame in a way that was not foreseen. The epidemic has produced a complex and urgent public health dilemma in a world that is fatigued by the COVID-19 pandemic. This fatigue, complicated social issues and a disease that has many scientific gaps and unknowns pose real risks for attempts to disrupt the transmission of MPXV during the 2022 multicountry outbreak. Intensified public health measures are urgently required, including equity and access to vaccination for those at risk. A key question is whether MPX, as a disease that is now transmitted from human to human rather than as a zoonosis, is here to fill the ‘vacated niche’.

## References

[1] Bray M. Keeping an eye on poxviruses. Am J Trop Med Hyg. 2009;80(4):499–500. 10.4269/ajtmh.2009.80.499 19346364

[2] Patel A, Bilinska J, Tam JC, et al. Clinical features and novel presentations of human monkeypox in a central London centre during the 2022 outbreak: Descriptive case series. BMJ. 2022;378:e072410. 10.1136/bmj-2022-072410 35902115 PMC9331915

[3] Marennikova SS, Šeluhina EM, Mal’Ceva NN, Cimiskjan KL, Macevic GR. Isolation and properties of the causal agent of a new variola-like disease (monkeypox) in man. Bull World Health Organ. 1972;46(5):599–611. 4340219 PMC2480798

[4] Lloyd-Smith JO. Vacated niches, competitive release and the community ecology of pathogen eradication. Phil Trans Royal Soc Biol Sci. 2019;368(1623):20120150. 10.1098/rstb.2012.0150 PMC3720048 23798698

[5] Beer EM, Rao VB. A systematic review of the epidemiology of human monkeypox outbreaks and implications for outbreak strategy. PLoS Negl Trop Dis. 2019;13(10):e0007791. 10.1371/journal.pntd.0007791 31618206 PMC6816577

[6] Reed KD, Melski JW, Graham MB, et al. The detection of monkeypox in humans in the Western Hemisphere. N Engl J Med. 2004;350(4):342–350. 10.1056/NEJMoa032299 14736926

[7] Di Giulio DB, Eckburg PB. Human monkeypox: An emerging zoonosis. Lancet Infect Dis. 2004;4(1):15–25. 10.1016/S1473-3099(03)00856-9 14720564 PMC9628772

[8] Tay JY, Vasoo S, Leo YS. Of migration, macaques, mice and men. Singapore Med J. 2021;62(11):562. 10.11622/smedj.2020010 31989182 PMC8804412

[9] World Health Organization. Multi-country outbreak of monkeypox. External situation report #4–24 August [homepage on the Internet]. 2022 [cited 2022 Aug 26]. Available from: https://www.who.int/publications/m/item/multi-country-outbreak-of-monkeypox--external-situation-report--4---24-august-2022

[10] Bragazzi NL, Kong JD, Mahroum N, et al. Epidemiological trends and clinical features of the ongoing monkeypox epidemic: A preliminary pooled data analysis and literature review. J Med Virol. 2022. 10.1002/jmv.27931 35692117

